# Tumor Microvessels with Specific Morphology as a Prognostic Factor in Esophageal Squamous Cell Carcinoma

**DOI:** 10.1245/s10434-025-17747-2

**Published:** 2025-07-10

**Authors:** Hnin Thida Tun, Masayoshi Fujisawa, Toshiaki Ohara, Seitaro Nishimura, Tomoyoshi Kunitomo, Kazuhiro Noma, Akihiro Matsukawa

**Affiliations:** 1 Department of Pathology and Experimental Medicine, Graduate School of Medicine, Dentistry and Pharmaceutical Sciences, Okayama University, Okayama, Japan; 2 Department of Gastroenterological Surgery, Graduate School of Medicine, Dentistry and Pharmaceutical Sciences, Okayama University, Okayama, Japan

**Keywords:** Esophageal neoplasms, Angiogenesis, Microvessel density, Pericytes, VEGF-A, Immunohistochemistry, Prognosis

## Abstract

**Background:**

Angiogenesis is essential for tumor progression. Microvessel density (MVD) is a widely used histological method to assess angiogenesis using immunostained sections, but its prognostic significance in esophageal cancer remains controversial. Recently, the evaluation of microvascular architecture has gained importance as a method to assess tumor aggressiveness. The present study aimed to identify the histological characteristics of tumor microvessels that are associated with the aggressiveness of esophageal squamous cell carcinoma.

**Patients and Methods:**

A total of 108 esophageal squamous cell carcinoma tissues were immunohistochemically stained with blood vessel markers and angiogenesis-related markers, including CD31, alpha smooth muscle actin, vascular endothelial growth factor A (VEGF-A), CD206, and D2-40. MVD, microvessel pericyte coverage index (MPI), and tumor vascular morphology were evaluated by microscopy.

**Results:**

MVD was significantly associated with patient outcomes, whereas neither MPI nor VEGF-A expression throughout the tumor showed a significant correlation. In addition, the presence of blood vessels encircling clusters of tumor cells, termed C-shaped microvessels, and excessively branching microvessels, termed X-shaped microvessels, was significantly associated with poor prognosis. These vessel types were also correlated with clinicopathological parameters, including deeper invasion of the primary tumor, presence of lymph node metastasis, advanced pathological stage, and distant metastasis. Focal VEGF-A immunoexpression in tumor cells was higher in areas containing C-shaped or X-shaped microvessels compared with areas lacking these vessel morphologies.

**Conclusions:**

The data suggest that tumor microvessels with specific morphologies (C-shaped and X-shaped microvessels) may serve as a promising prognostic factor in esophageal squamous cell carcinoma.

**Supplementary Information:**

The online version contains supplementary material available at 10.1245/s10434-025-17747-2.

Esophageal cancer is the seventh most commonly diagnosed cancer and the sixth leading cause of cancer death globally.^1,2^ It imposes a significant clinical burden, particularly in Asia, due to its high morbidity and mortality.^1^ Despite recent advances in treatment and techniques,^3^ the prognosis remains poor, with a 5-year relative survival rate of only 22%.^4^

Angiogenesis, the complex process by which new blood vessels form from preexisting ones, is essential for tumor development.^5^ Vascular endothelial growth factor (VEGF) plays an essential role in tumor angiogenesis and regulates various functions of endothelial cells, including proliferation, migration, survival, and permeability.^6^ While VEGF is mainly secreted by tumor cells,^7,8^ tumor-associated macrophages (TAMs), particularly M2 macrophages, also contribute to its production.^9^ To microscopically evaluate the angiogenesis and the functional status of newly formed vessels, microvessel density (MVD) and microvessel pericyte coverage index (MPI) are widely used techniques.^10,11^ However, the prognostic significance of commonly used angiogenesis markers such as MVD, MPI, and VEGF expression in predicting patient outcomes remains controversial in many malignancies, including esophageal cancer.^12–17^

Unlike normal vasculature, tumor vasculature exhibits an irregular and disorganized structure.^18^Recently, several endoscopic studies have investigated the morphology of microvessels in esophageal cancer to predict the depth of tumor invasion.^19,20^ Moreover, previous research has reported that the formation of abnormally shaped blood vessels is associated with more aggressive tumor characteristics in undifferentiated sarcoma^21^ and gastric cancer.^22^ In addition, we identified two distinct microvessel morphologies, termed C-shaped and X-shaped microvessels, and demonstrated that their presence was associated with shorter patient survival in invasive breast cancer.^23^ However, the mechanisms underlying the formation of abnormally shaped blood vessels remain unclear, and the association between these vessel abnormalities and tumor aggressiveness in esophageal cancer requires further clarification. Therefore, the aim of this study was to identify the histological characteristics of tumor microvessels involved in the aggressiveness of esophageal squamous cell carcinoma.

## Patients and Methods

### Sample Collection

A total of 147 radical esophagectomy cases performed between January 2008 and December 2010 were consecutively retrieved from the pathology database of Okayama University Hospital. Of these, 39 cases were excluded for the following reasons: (1) absence of residual tumor cells following endoscopic submucosal dissection or neoadjuvant therapy, (2) histological type not squamous cell carcinoma or its variant, (3) carcinoma in situ (pTis), and (4) loss of invasive tumor tissue in paraffin blocks due to prior research use. Thus, 108 patients with invasive squamous cell carcinoma were included in the study. The detailed flow diagram is shown in Fig. S1. Among these patients, 15 patients (13.9%) received neoadjuvant chemotherapy, and 10 patients (9.3%) received neoadjuvant chemoradiotherapy. This study was approved by the Ethical Review Board of Okayama University (approval no. 1801-023; Okayama, Japan).

### Histopathological Analysis

For each case, a representative block containing the largest tumor tissue was selected. FFPE tissues were serially sectioned at a thickness of 4 µm for both hematoxylin and eosin (H&E) staining and immunohistochemical staining. Pathological stage was determined according to the eighth edition of the Union for International Cancer Control (UICC) TNM classification.

### Immunohistochemical Staining

A double immunohistochemical staining method was employed to simultaneously stain CD31 and other markers (αSMA, VEGF-A, CD206). CD31 was visualized in red with Vector Red, while αSMA, VEGF-A, or CD206 were visualized in brown using DAB. In addition, single staining for VEGF-A (brown) was performed separately. The sections were also stained with D2-40 (brown) to identify lymphatic vessels. Detailed information regarding the primary antibodies and the immunostaining procedures is provided in Tables S1, S2, and S3.

### Quantitative Evaluation of Tumor Microvessels

*MVD*: Although we performed CD31 and αSMA double staining, MVD was assessed solely on the basis of the red staining of CD31. MVD was calculated as the total number of microvessels in ten high-power fields (HPF) of the region with the highest vessel density (0.237 mm^2^ per 1 HPF).^10^ The median MVD value across all the cases was used as the cutoff value to classify tumors as having low or high MVD.

*MPI*: If CD31-positive microvessels were partially or completely rimmed by αSMA-positive cells, they were considered to accompany pericytes. MPI was calculated as the percentage of microvessels with pericytes relative to the total number of CD31-positive microvessels in the same ten HPFs used for MVD assessment.^11,24^ Cases were then classified into low or high MPI groups on the basis of the median MPI value.

### Morphology of Tumor Microvessels

The microscopic identification of microvessels with abnormal morphology was performed according to the criteria slightly modified from our previous study.^23^ Although CD31 and αSMA double staining was applied, we focused solely on the red staining of CD31 to identify the specified microvessel morphologies. These structures were considered positive if they were present throughout the representative section. Two pathologists (H.T.T. and M.F.) independently assessed the slides, and any ambiguous cases were discussed until consensus was reached.

*C-shaped microvessel*: A microvessel was defined as C-shaped if it met the following two criteria: (1) the presence of tumor cells encircled by an intratumoral microvessel, with or without a lumen, that forms the appearance of the letter “C”, with encircling defined as the angle formed by lines extending from each tip of the blood vessel to the nucleus of the innermost tumor cell, which must be less than 90 degrees and (2) the distance across the blood vessel, measured as a straight line connecting the two tips, should not exceed 200 micrometers.

*X-shaped microvessel*: A microvessel was defined as X-shaped if it met the following two criteria: (1) an intratumoral microvessel with four or more simultaneous branches, forming the appearance of the letter “X” and (2) at least one branch must be in close proximity to tumor cells.

### Evaluation of VEGF-A Immunoexpression

VEGF-A expression throughout the invasive tumor area was assessed using single staining following previously published methods.^25,26^ Briefly, a score ranging from 0 to 12 was calculated by multiplying the percentage of positively stained tumor cells (0 = 0; 1–10% = 1; 11–50% = 2; 51–80% = 3; 81–100% = 4) by the staining intensity (negative = 0; weak = 1; moderate = 2; strong = 3). Cases were subsequently classified into two groups: VEGF-A low (score 0–3) and VEGF-A high (score 4–12). The focal VEGF-A staining intensity around the specific microvessels was evaluated using microscopic images of the double-stained sections (CD31 and VEGF-A) that were captured at 400× magnification (0.237 mm^2^ per 1 HPF) using an Olympus BX43 optical microscope equipped with a DP73 digital camera (Olympus, Tokyo, Japan). Camera settings were controlled using CellSens Standard software (Olympus). Image analysis was performed using the IHC profiler plugin in ImageJ software (version 1.54h), following a previously established methodology.^27^ The regions of interest (ROIs) for the analysis of VEGF-A staining intensity were constituted of all tumor cells surrounding various configurations of blood vessels within the full microscopic field at 400× magnification and were selected manually after color deconvolution. VGEF-A staining intensity was measured as the “mean gray value” and expressed as DAB reciprocal staining intensity (RSI = 255, mean gray value). Only the cases with C-shaped or X-shaped microvessels were included in this analysis. The average value of VEGF-A intensity in tumor cells around C-shaped or X-shaped microvessels was compared with that of corresponding control areas. The control areas were randomly selected from five or six areas with regularly shaped microvessels in the same section, as five or six were the maximum number of C-shaped or X-shaped microvessels in one section, respectively.

### M2 Macrophage Count around Microvessels

CD206 is one of the common markers for M2 tumor-associated macrophages (TAMs).^28^ CD31 and CD206 double staining was performed to assess endothelial cells and M2 macrophages simultaneously. The average number of M2 macrophages per HPF in regions containing C-shaped or X-shaped microvessels was compared with that of control areas in the same section. The control areas were determined similarly to the VEGF-A analysis.

### Statistical Analysis

Statistical analysis was performed using GraphPad Prism software (version 6.0d) and EZR software (version 1.55). Fisher’s exact test or Pearson’s chi-squared test was used to determine the association between blood vessel parameters (MVD and MPI), whole tumor VEGF-A, and clinicopathological factors of patients. Paired focal VEGF-A staining intensity and paired M2 macrophage counts were analyzed using the Wilcoxon matched-pairs signed-rank test. The 5-year relapse-free survival (RFS), 5-year cancer-specific survival (CSS), and 5-year overall survival (OS) outcomes were evaluated by Kaplan–Meier analysis (log-rank test). Multivariate survival analyses were performed using Cox proportional hazard regression. Survival was defined as the time from the date of the esophagectomy procedure to the date of recurrence (RFS), or to the date of death from esophageal cancer (CSS), or to the date of death (OS). A two-tailed *p*-value < 0.05 was considered statistically significant.

## Results

### MVD and MPI and Their Correlation with Clinicopathological Factors and Survival in Esophageal Squamous Cell Carcinoma

**Fig. 1:**
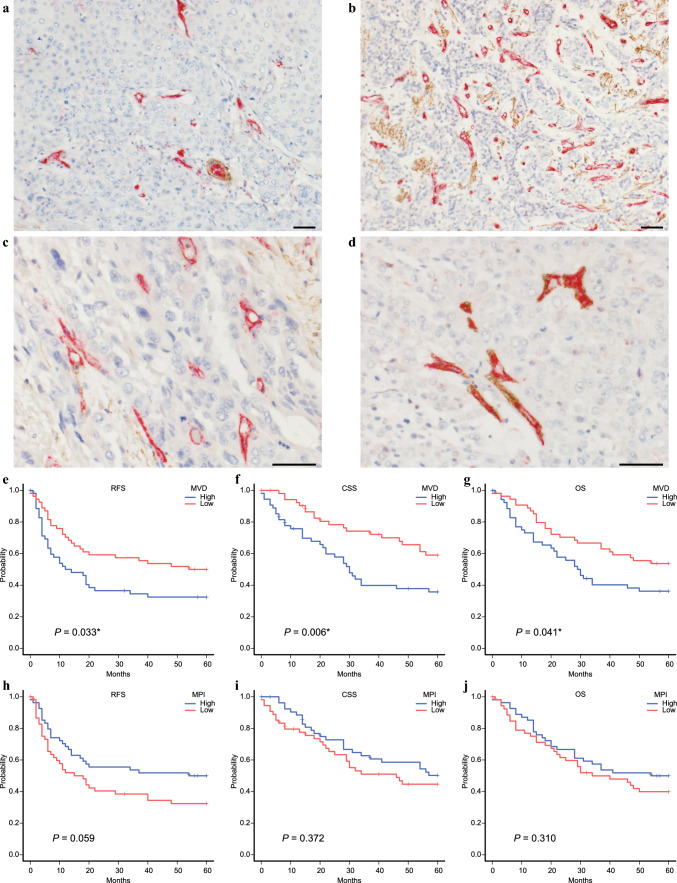
Evaluation of MVD and MPI and their association with prognosis; blood vessel endothelial cells were stained red (CD31), and pericytes, fibroblasts, and smooth muscle cells were stained brown (αSMA) by double staining; red staining of CD31 alone is sufficient to determine MVD; **A** MVD low; **B** MVD high; **C** MPI low; **D** MPI high; **E**–**G** Kaplan–Meier analysis for 5-year relapse-free survival (RFS), cancer-specific survival (CSS), and overall survival (OS) with MVD in 108 cases of esophageal cancer; **H**–**J** Kaplan–Meier analysis for 5-year RFS, CSS, and OS with MPI in 108 cases of esophageal cancer; scale bars indicate 50 μm

MVD and MPI, two key angiogenesis parameters, were analyzed in 108 invasive esophageal cancer tissue samples. MVD, defined as the number of microvessels in 10 HPFs, and the endothelial cells of the microvessels were stained with CD31 antibody (Fig. 1A, B). The MVD values ranged from 7 to 123, with a median of 42.5 (mean 43.7, SD 23). MPI, an indicator of blood vessel maturity, was defined as the percentage of microvessels that have pericytes relative to the total number of microvessels (Fig. 1C, D). MPI values ranged from 2 to 71%, with a median of 18% (mean 22%, SD 0.1%). The relationships between microvessel parameters (MVD and MPI) and patients’ clinicopathological factors are summarized in Table 1. High MVD and low MPI were significantly correlated with deeper invasion of the primary tumor (MVD and MPI: *p* < 0.001) and advanced pathological stage (MVD: *p* = 0.033, MPI: *p* = 0.012). High MVD was significantly associated with poor 5-year RFS, CSS, and OS (*p* = 0.033, *p* = 0.006, and* p* = 0.041, Fig. 1E–G), whereas MPI showed no significant association with 5-year RFS, CSS, and OS (*p* = 0.059, *p* = 0.372, and *p* = 0.310, respectively, Fig. 1H–J). Although high MVD and low MPI were linked to advanced tumors, only MVD, but not MPI, was related to poor prognosis in this study.

**Table 1:** Correlation between microvessel parameters and patients’ clinicopathological factors

Clinicopathological factors	Total *N*	MVD			MPI		
		Low (*n* = 54)	High (*n* = 54)	*p*-value	Low (*n* = 54)	High (*n* = 54)	*p*-value
*Patients’ age*							
< 65 years	41	24	17	0.234	21	20	1.000
≥ 65 years	67	30	37		33	34	
*Gender*							
Male	93	46	47	1.000	47	46	1.000
Female	15	8	7		7	8	
*T, Primary tumor*							
T1–T2	53	39	14	< 0.001*	17	36	< 0.001*
T3–T4	55	15	40		37	18	
*N, Regional lymph nodes*							
N0	50	28	22	0.335	21	29	0.176
N1–N3	58	26	32		33	25	
*M, Distant metastasis*							
M0	103	52	51	1.000	50	53	0.363
M1	5	2	3		4	1	
*Pathological stage*							
I–II	58	35	23	0.033*	22	36	0.012*
III–IV	50	19	31		32	18	
*Differentiation*							
Well	23	12	11	0.189	12	11	0.871
Moderate	59	33	26		28	31	
Poor	26	9	17		14	12	
*Lymphatic vessel invasion*							
Yes	30	16	14	0.830	12	18	0.283
No	78	38	40		42	36	
*Treatment prior to surgery*							
None	83	43	40	0.649	39	44	0.362
Neoadjuvant	25	11	14		15	10	

*p < 0.05.

*Irregularly Shaped* (*C-shaped and X-shaped*) *Microvessels and Their Correlation with Clinicopathological Factors and Survival in Esophageal Squamous Cell Carcinoma*

**Fig. 2:**
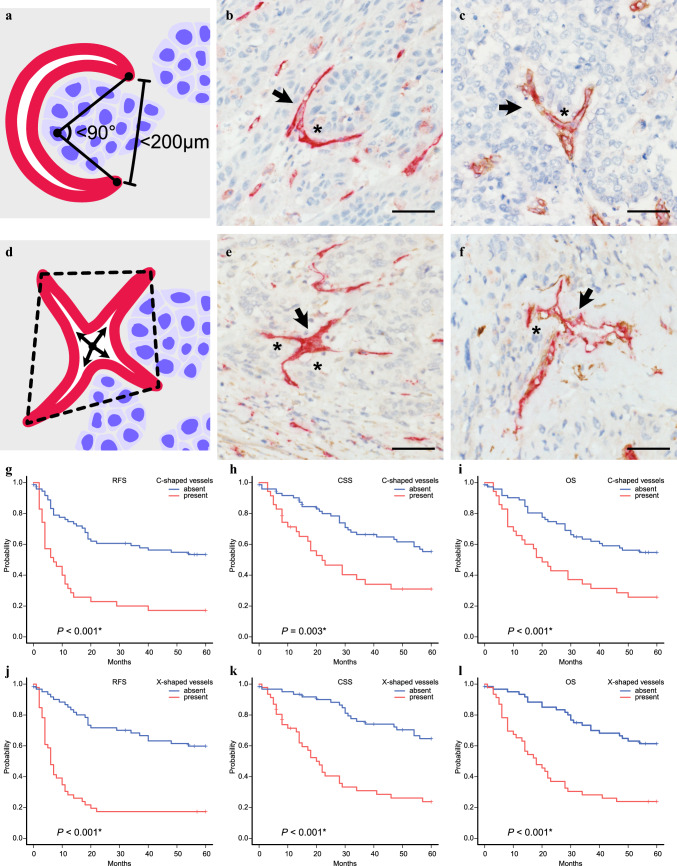
Irregularly shaped microvessels and their association with prognosis; although double staining of CD31 (red) and αSMA (brown) was used, CD31 (red) staining alone was sufficient to identify the C-shaped and X-shaped microvessels; **A** schematic illustration of a C-shaped microvessel (red), it must enclose a cluster of tumor cells (blue); **B** photomicrographic image of a C-shaped microvessel (arrow) encircling the tumor cells (asterisk), αSMA staining was completely absent around this C-shaped microvessel; **C** another C-shaped microvessel accompanied pericytes (brown); **D** schematic illustration of an X-shaped microvessel (red), it must have four simultaneous branches and accompany tumor cells nearby (within the polygon); **E** photomicrographic image of an X-shaped microvessel (arrow) accompanying nearby tumor cells (asterisks), αSMA staining was completely absent around the X-shaped microvessel; **F** another example of an X-shaped microvessel with pericytes (brown); **G**–**I** Kaplan–Meier analysis for 5-year RFS, CSS, and OS with C-shaped microvessels in 108 cases of esophageal cancer; **J**–**L** Kaplan–Meier analysis for 5-year RFS, CSS, and OS with X-shaped microvessels in 108 cases of esophageal cancer; scale bars indicate 50 μm

The characteristics of irregularly shaped microvessels in esophageal cancer were evaluated by light microscopy. C-shaped microvessels were defined as microvessels encircling clusters of tumor cells (Fig. 2A) and were identified in 35 out of 108 cases (32.4%). The majority of C-shaped microvessels did not accompany pericytes (Fig. 2B), while a minority had pericytes (12%, Fig. 2C), a slightly lower proportion than the average of total vessels examined (19%). X-shaped microvessels were defined as excessively branching microvessels with four or more branches present simultaneously (Fig. 2D). X-shaped microvessels were identified in 46 out of 108 cases (42.6%). Most X-shaped microvessels were not covered by pericytes (Fig. 2E), and the pericyte coverage index was 10% (Fig. 2F), also lower than the total vessels (19%). Both C-shaped and X-shaped microvessels were found within the tumor but rarely seen at the peripheral margins of the tumor. The relationships between the irregularly shaped microvessels and patients’ clinicopathological factors are summarized in Table 2. The presence of C-shaped and X-shaped microvessels was significantly associated with deeper invasion of the primary tumor (C-shaped and X-shaped: *p* < 0.001), presence of regional lymph node metastasis (C-shaped: *p* = 0.004, X-shaped:* p* = 0.002), advanced pathological stage (C-shaped and X-shaped: *p* < 0.001), high MVD (C-shaped: *p* = 0.039, X-shaped: *p* < 0.001), and low MPI (C-shaped: *p* = 0.001, X-shaped: *p* = 0.032). Additionally, the presence of X-shaped microvessels was correlated with the presence of distant metastasis at the time of diagnosis (*p* = 0.012). However, no significant relationships were found between C-shaped or X-shaped microvessels and patients’ age, gender, tumor cell differentiation, or lymphatic vessel invasion. The presence of C-shaped microvessels was significantly associated with poor 5-year RFS, CSS, and OS (*p* < 0.001, *p* = 0.003, and *p* < 0.001, respectively, Fig. 2G–I). The presence of X-shaped microvessels also demonstrated a significant correlation with worsened 5-year RFS, CSS, and OS (*p* < 0.001, Fig. 2J–L). Both C-shaped and X-shaped microvessels also negatively impacted survival in patients who did not receive neoadjuvant therapy (*n* = 83) (Fig. S2A–F). Multivariate survival analysis was performed to evaluate the prognosis prediction of MVD, C-shaped and X-shaped microvessels, and established prognostic factors. As presented in Table 3, the presence of X-shaped microvessels, but not MVD, was an independent prognostic factor for 5-year RFS, CSS, and OS. The presence of C-shaped microvessels was found to be an independent prognostic factor for 5-year RFS only. These findings suggest that these irregularly shaped microvessels, especially X-shaped, strongly predict poor prognosis in esophageal cancer.

**Table 2:** Relationship between irregularly shaped microvessels and patients’ clinicopathological factors

Clinicopathological factors	Total *N*	C-shaped blood vessels			X-shaped blood vessels		
		Absent (*n* = 73)	Present (*n* = 35)	*p*-value	Absent (*n* = 62)	Present (*n* = 46)	*p*-value
*Patients’ age*							
< 65 years	41	30	11	0.400	27	14	0.229
≥ 65 years	67	43	24		35	32	
*Gender*							
Male	93	61	32	0.377	50	43	0.089
Female	15	12	3		12	3	
*T, Primary tumor*							
T1–T2	53	46	7	< 0.001*	45	8	< 0.001*
T3–T4	55	27	28		17	38	
*N, Regional lymph nodes*							
N0	50	41	9	0.004*	37	13	0.002*
N1–N3	58	32	26		25	33	
*M, Distant metastasis*							
M0	103	71	32	0.326	62	41	0.012*
M1	5	2	3		0	5	
*Pathological stage*							
I–II	58	49	9	< 0.001*	44	14	< 0.001*
III–IV	50	24	26		18	32	
*Differentiation*							
Well	23	14	9	0.389	12	11	0.753
Moderate	59	43	16		36	23	
Poor	26	16	10		14	12	
*MVD*							
Low	54	42	12	0.039*	45	9	< 0.001*
High	54	31	23		17	37	
*MPI*							
Low	54	28	26	0.001*	25	29	0.032*
High	54	45	9		37	17	
*Lymphatic vessel invasion*							
Yes	30	18	12	0.360	15	15	0.388
No	78	55	23		47	31	
*Treatment prior to surgery*							
None	83	65	18	< 0.001*	56	27	< 0.001*
Neoadjuvant	25	8	17		6	19	

*p < 0.05.

**Table 3:** Multivariate survival analysis with pathological stage, lymphatic vessel invasion (LVI), MVD, C-shaped microvessels, and X-shaped microvessels

Explanatory variable	5-year RFS		5-year CSS		5-year OS	
	HR (95% CI)	*p-*value	HR (95% CI)	*p*-value	HR (95% CI)	*p-*value
Pathological stage (III–IV versus I–II)	1.926 (1.082–3.429)	0.026*	1.849 (0.990–3.454)	0.054	1.578 (0.872–2.854)	0.132
LVI (present versus absent)	1.062 (0.587–1.890)	0.838	1.435 (0.78–2.618)	0.239	1.205 (0.673–2.159)	0.530
MVD (high versus low)	0.783 (0.427–1.437)	0.430	1.198 (0.622–2.306)	0.589	0.939 (0.507–1.742)	0.842
C-shaped (present versus absent)	1.845 (1.055–3.228)	0.032*	1.207 (0.658–2.214)	0.543	1.359 (0.758–2.435)	0.303
X-shaped (present versus absent)	3.423 (1.740–6.735)	< 0.001*	2.677 (1.308–5.480)	0.007*	2.713 (1.362–5.402)	0.005*

*HR* hazard ratio, *CI* confidence interval

*p < 0.05.

### VEGF-A Immunoexpression in the Entire Tumor Area and Focal Tumor Area Surrounding the Specific Morphology of Tumor Microvessels

VEGF-A is one of the most important angiogenesis factors, and its expression has been histologically evaluated as a semiquantitative score on the basis of the proportion of positive cells and staining intensity throughout the tumor area. VEGF-A staining was observed in the cytoplasm of both tumor cells and stromal cells, with heterogeneous staining intensity within the tumor area. Among our 108 cases, 65 (60.2%) were classified as VEGF-A low and 43 (39.8%) as VEGF-A high (Fig. S3A, B). VEGF-A expression throughout the tumor area was not associated with any clinicopathological parameters, including the presence of C-shaped and X-shaped microvessels (Table S4). VEGF-A showed no significant association with 5-year RFS, CSS, and OS (*p* = 0.483, *p* = 0.656, and *p* = 0.692, respectively, Fig. S3C–E). These data suggest that VEGF-A throughout the tumor does not account for the pathogenesis of the abnormal microvessels.

**Fig. 3:**
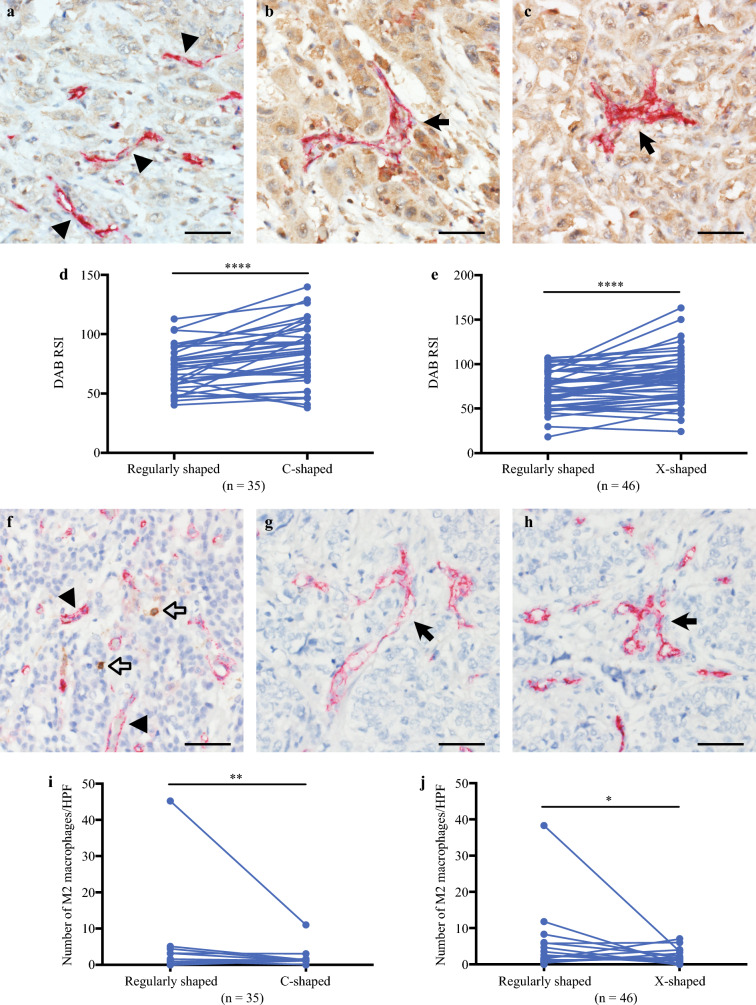
Immunoexpression of focal VEGF-A in tumor cells and the number of M2 macrophages around tumor microvessels, blood vessel endothelial cells were stained red (CD31), tumor cells were stained brown (VEGF-A), and M2 macrophages were also stained brown (CD206) by double staining; **A** tumor cells around regularly shaped microvessels (arrowheads) showed low VEGF-A staining intensity; **B**, **C** tumor cells around C-shaped (**B**) and X-shaped (**C**) microvessels (arrows) showed higher VEGF-A staining intensity than **A**; **D**, **E** paired focal VEGF-A staining intensity in tumor cells surrounding regularly shaped blood vessels and C-shaped blood vessels or X-shaped blood vessels, DAB staining intensities were measured as “mean gray value” and expressed as reciprocal staining intensities (RSI = 255, mean gray value), ****p < 0.001; **F** M2 macrophages (open arrows) around regularly shaped microvessels (arrowheads); **G**, **H** M2 macrophages were not detected around either the C-shaped microvessel or the X-shaped microvessel (arrow); **I**, **J** paired numbers of M2 macrophages around regularly shaped microvessels and C-shaped microvessels or X-shaped microvessels, **p = 0.005, *p = 0.042; scale bars indicate 50 μm

Given the heterogeneous staining intensity of intratumoral VEGF-A, we next focused on focal VEGF-A immunoexpression around both normal and abnormal microvessels. The intensity of VEGF-A staining was significantly higher in tumor cells surrounding C-shaped microvessels compared with those adjacent to regularly shaped microvessels (*n* = 35, *p* < 0.001). Similarly, tumor cells adjacent to X-shaped microvessels exhibited significantly greater VEGF-A staining intensity compared with those around regularly shaped microvessels (*n* = 46, *p* < 0.001), as illustrated in Fig. 3A–E. Although most of the cells with VEGF-A positivity seemed to be tumor cells, M2 macrophages are also reported to be a source of VEGF-A. To determine the contribution of macrophages to VEGF production around microvessels, the number of M2 macrophages around the microvessels was measured. M2 macrophages were present around regularly shaped microvessels, while they were rarely detected around C-shaped and X-shaped microvessels. The number of M2 macrophages was lower around C-shaped microvessels than around regularly shaped microvessels (*n* = 35, *p =* 0.005). The number of M2 macrophages around X-shaped microvessels was also lower than that around regularly shaped microvessels (*n* = 46, *p* = 0.042), as shown in Fig. 3F–J. These findings suggest that the elevated level of VEGF-A expression, which originates from tumor cells, not macrophages, plays a critical role in the formation of abnormally shaped microvessels.

## Discussion

MVD has been widely considered a predictor of tumor growth and patient outcomes. Numerous studies have reported a significant correlation between high MVD and unfavorable prognosis in esophageal cancer.^29–38^ However, some studies have shown no significant correlation between MVD and prognosis,^39,40^ and a few studies have even found that high MVD is associated with better survival.^41,42^ MPI has been known as an indicator that can assess the maturity of blood vessels. One study mentioned that low MPI is correlated with poor prognosis in colorectal cancer,^16^ but another study proved otherwise in breast cancer.^17^ To date, no clinical studies have investigated the relationship between MPI and patient outcomes in esophageal cancer. In our study, we found that MVD was associated with prognosis, whereas MPI was not. The discrepancies regarding the prognostic significance of MVD and MPI among the previous studies are likely due to the wide range of variables in the selection of primary antibodies to detect microvessels, areas of hot spots, magnification for counting individual microvessels, and cutoff points. Quantitatively counting the number of tumor microvessels (MVD) may not be sufficient to determine the functional abnormalities of intratumoral microvessels because they exhibit structural complexity in the advanced stage of tumors,^11,43,44^ therefore, attention must be paid to the morphology of the microvessels.

On the basis of the architectural pattern of microvessels, we previously introduced two distinct types of microvessels in invasive breast cancer: C-shaped and X-shaped microvessels.^23^ As shown in this study, C-shaped vessels were occasionally observed, while X-shaped microvessels were more frequently detected in esophageal cancer. Our findings indicate that irregular blood vessel morphologies were significantly correlated with deeper invasion of the primary tumor, presence of regional lymph node metastasis, advanced pathological stage, and poor prognosis. Endoscopic studies of esophageal cancer have shown that tumor vessels develop structural irregularities as tumor grade progresses, from noncancerous and precancerous lesions to carcinoma.^19,20^ Microscopically, a study of laryngeal tumor described that irregularly shaped vasculature was prominent in the high-grade (poorly differentiated) squamous cell carcinoma.^44^ Our study demonstrated that irregular tumor microvessels are present across any stage of invasive tumors but are more likely to be found in advanced stages of esophageal cancer, further supporting the idea that irregular microvessel morphology is associated with tumor aggressiveness.

Of these two specific microvessels, X-shaped microvessels seem to be the better prognostic factor than C-shaped microvessels, as only the former showed a significant correlation with the presence of distant metastasis and identified as an independent prognostic factor, separate from tumor stage, in multivariate analysis. This trend is consistent with the results of our previous study using breast cancer.^23^ Our previous three-dimensional (3D) analysis revealed that these two microvessels tend to appear close to each other, suggesting that they are different aspects of a similar phenomenon.^23^ X-shaped microvessels are more easily identifiable under two-dimensional cross-sections than C-shaped microvessels; therefore, they may serve as a better indicator of abnormal microvessels potentially associated with tumor aggressiveness.

The alteration in vascular morphology and its correlation with poor prognosis may be due to the inappropriate pericyte coverage of tumor microvessels, as pericytes tend to stabilize blood vessels and negatively impact on angiogenesis, vessel branching, vascular permeability, and tumor cell infiltration into the vascular lumen.^45,46^ In the tumor vasculature, pericytes are loosely attached to the basement membrane of the endothelial cells; partial or even complete absence of pericytes has been found in tumor microvessels, contributing to the increased metastatic potential of the tumor.^45–47^ In our study, we found that the majority of C-shaped and X-shaped microvessels were not covered by pericytes, i.e., αSMA expression was completely absent in the entire circumference of peri-endothelial cells, indicating the absence or immaturity of the pericyte lining of these two vessels; therefore, tumor cells can easily penetrate into the microvessels and subsequently have a high possibility of obtaining distant metastasis. Both C-shaped and X-shaped microvessels were observed within the tumor but rarely seen at the peripheral margins of the tumor. Similarly, the pericyte coverage of vessels in the center of the tumor was reported to be lower than the periphery of the tumor in gastrointestinal carcinomas.^48^ This suggests that variability of pericyte enrichment may affect the morphology and functional status of the microvessels.

To understand why C-shaped and X-shaped microvessels form in aggressive tumors, we investigated the role of VEGF-A, as it is a potent mediator of neovascularization; therefore, it may be responsible for forming abnormal, tortuous, and immature blood vessels.^18^ Endothelial cell survival depends mainly on VEGF stimulation when vessels are not covered by pericytes.^45^ Despite the above findings, the prognostic role of VEGF-A expression in the entire tumor area remains controversial,^26,34,49–54^ and our study did not support its significance as a prognostic factor. In contrast, our study found that focal VEGF-A staining intensity in tumor cells around C-shaped and X-shaped microvessels was significantly higher than that around regularly shaped microvessels. These data highlight the importance of considering the intratumoral heterogeneity of VEGF-A. However, the focal VEGF-A staining intensity did not differ between tumor cells adjacent to C-shaped and X-shaped microvessels (Fig. S4), suggesting that these morphological differences were not controlled by VEGF expression levels. The mechanisms underlying the formation of these irregular microvessels warrants further investigation.

M2 macrophages also support angiogenesis by secreting VEGF to promote tumor growth.^55^ However, our findings indicated that M2 macrophages are not located around the C-shaped and X-shaped microvessels. This suggests that the formation of C-shaped and X-shaped microvessels is mediated by tumor-derived VEGF-A and not by M2 macrophages. Because angiogenesis is a complex process, further studies are needed to determine whether additional factors contribute to the formation of abnormally shaped microvessels.

Another finding was that most of the C-shaped and X-shaped microvessels appeared to have collapsed vascular lumens compared with peritumoral vessels. This may be due to the presence of high interstitial pressure, as solid tumors typically have increased interstitial fluid pressure that is uniformly elevated within the tumor mass due to pathological changes in the lymphatic vessels.^56^

Our findings may be clinically useful for guiding treatment decisions and developing new therapeutic strategies. Although VEGF inhibitors such as bevacizumab are widely used in various cancers, their efficacy in esophageal cancer remains unclear. These agents have not demonstrated benefit in adenocarcinoma, and evidence for their use in squamous cell carcinoma is limited.^57–59^ Suppression of VEGF may be effective if the treatment is limited to patients with C-shaped and X-shaped microvessels, particularly in squamous cell carcinoma. Future studies with larger cohorts and prospective designs are warranted to validate whether the presence of abnormal vessels can effectively guide treatment decisions.

To our knowledge, this is the first study to microscopically analyze the role of intratumoral vascular morphology in esophageal squamous cell carcinoma. Our results suggest that, in addition to high MVD, the presence of microvessels with specific morphologies (C-shaped or X-shaped) serves as an indicator of adverse outcomes. Notably, X-shaped microvessels appear to be a strong prognostic factor independent of tumor stage. These findings of abnormal vessel structures are in concordance with our previous study of invasive breast cancer, suggesting their potential relevance across multiple tumor types.

## Supplementary Information

Supplementary file 1
