# Supplementary material for: Tumor Microvessels with Specific Morphology as a Prognostic Factor in Esophageal Squamous Cell Carcinoma
Source: Ann Surg Oncol. 2025 Jul 10;32(10):8024–36. doi: 10.1245/s10434-025-17747-2 (PMC12454593; doi:10.1245/s10434-025-17747-2)
Supplement: Supplementary file 1 [file 10434_2025_17747_MOESM1_ESM.pdf]

Table S1. List of primary antibodies

Primary antibody	Clone	Pretreatment	Dilution	Incubation time	Manufacturer
CD31[a]	89C2	EDTA (pH 8.0)	1:1000	Overnight at 4°C	Cell Signaling, USA
CD31[b]	EPR3094	EDTA (pH 8.0)	1:250	2 hours at RT	Abcam, UK
αSMA	Ab5694	EDTA (pH 8.0)	1:400	1 hour at RT	Abcam, UK
VEGF-A	VG-1	EDTA (pH 8.0)	1:500	Overnight at 4°C	Abcam, UK
Podoplanin	D2-40	EDTA (pH 8.0)	1:200	Overnight at 4°C	Dako, USA
CD206	#24595	EDTA (pH 8.0)	1:600	2 hours at RT	Cell Signaling, USA

[a]Used for CD31 and αSMA double staining or CD31 and CD206 double staining.

[b]Used for CD31 and VEGF-A double staining.

Table S2. Double immunohistochemical staining procedure for CD31 and αSMA or VEGF-A or CD206

Step	Process
1	Deparaffinization of slides in xylene, followed by 100%, 90% and 80% ethanol
2	Rinse the slides with water
3	Enclose sections in a pressure cooker with 0.5 M EDTA (pH 8.0) and microwave (700W) for 20 minutes
4	Cool naturally[a]
5	First primary antibody incubation[a] (CD31)
6	Secondary antibody[b] incubation for 60 minutes[a]
7	Histochemical reaction with ALP[c]
8	Rinse with Tris-buffered saline with Tween 20, three times
9	Incubate the slides in 10% H_2O_2 for 10 minutes[a]
10	Protein block[d] for 15 minutes
11	Second primary antibody incubation[a] (αSMA or VEGF-A or CD206)
12	Secondary antibody incubation (simple stain MAX-PO, Nichirei, Tokyo, Japan) for 60 minutes[a]
13	Histochemical reaction with DAB[e]
14	Counterstaining with hematoxylin
15	Dry slides on a hot plate (45 °C) for 20 minutes
16	Mount sections by a conventional method

[a]Followed by a rinse with Tris-buffered saline with Tween 20, three times.

[b]ImmPRESS®-AP Horse Anti-Rabbit/Anti-Mouse IgG Polymer Detection Kit, Alkaline Phosphatase (MP-5401/MP-5402), Vector, USA.

[c]ImmPACT® Vector® Red Substrate Kit, Alkaline Phosphatase (AP) SK-5105, Vector, USA.

[d]Protein Block Serum-Free (Code X0909), Dako, USA.

[e]Liquid DAB+ Substrate Chromogen System (Code K3468), Dako, USA.

Table S3. Single immunohistochemical staining procedure for D2-40 and VEGF-A

Step	Process
1	Deparaffinization of slides in xylene, followed by 100%, 90% and 80% ethanol
2	Rinse the slides with water
3	Enclose sections in a pressure cooker with 0.5 M EDTA buffer (pH 8.0) and microwave (700W) for 20 minutes
4	Cool naturally[a]
5	Incubate the slides in 10% H_2O_2 for 10 minutes[a]
6	Primary antibody incubation[a]
7	Secondary antibody incubation (simple stain MAX-PO, Nichirei, Tokyo, Japan) for 60 minutes[a]
8	Histochemical reaction with DAB[b]
9	Counterstaining with hematoxylin
10	Dehydration in 80%, 90% and 100% ethanol and clearing in xylene
11	Mount sections by a conventional method

[a]Followed by a rinse with Tris-buffered saline with Tween 20, three times.

[b]Liquid DAB+ Substrate Chromogen System (Code K3468), Dako, USA.

Table S4. Association between whole tumor VEGF-A expression and patients' clinicopathological factors including the specific morphology of microvessels

Clinicopathological factors	Total N	VEGF-A		
		Low (n=65)	High (n=43)	*P*
Patients' Age				
<65 years	41	25	16	1.000
≥65 years	67	40	27	
Gender				
Male	93	58	35	0.269
Female	15	7	8	
T – Primary tumor				
T1 - T2	53	30	23	0.556
T3 - T4	55	35	20	
N – Regional lymph nodes				
N0	50	31	19	0.844
N1 -N3	58	34	24	
M – Distant metastasis				
M0	103	61	42	0.646
M1	5	4	1	
Pathological stage				
I - II	58	37	21	0.436
III - IV	50	28	22	
Differentiation				
Well	23	14	9	0.451
Moderate	59	38	21	
Poor	26	13	13	
MVD				
Low	54	32	22	1.000
High	54	33	21	
MPI				
Low	54	35	19	0.432
High	54	30	24	
Lymphatic vessel invasion				
Yes	30	15	15	0.195
No	78	50	28	
Treatment prior to surgery				
None	83	48	35	0.485
Neoadjuvant	25	17	8	
C-shaped microvessel				
Absent	73	46	27	0.408
Present	35	19	16	
X-shaped microvessel				
Absent	62	34	28	0.234
Present	46	31	15	

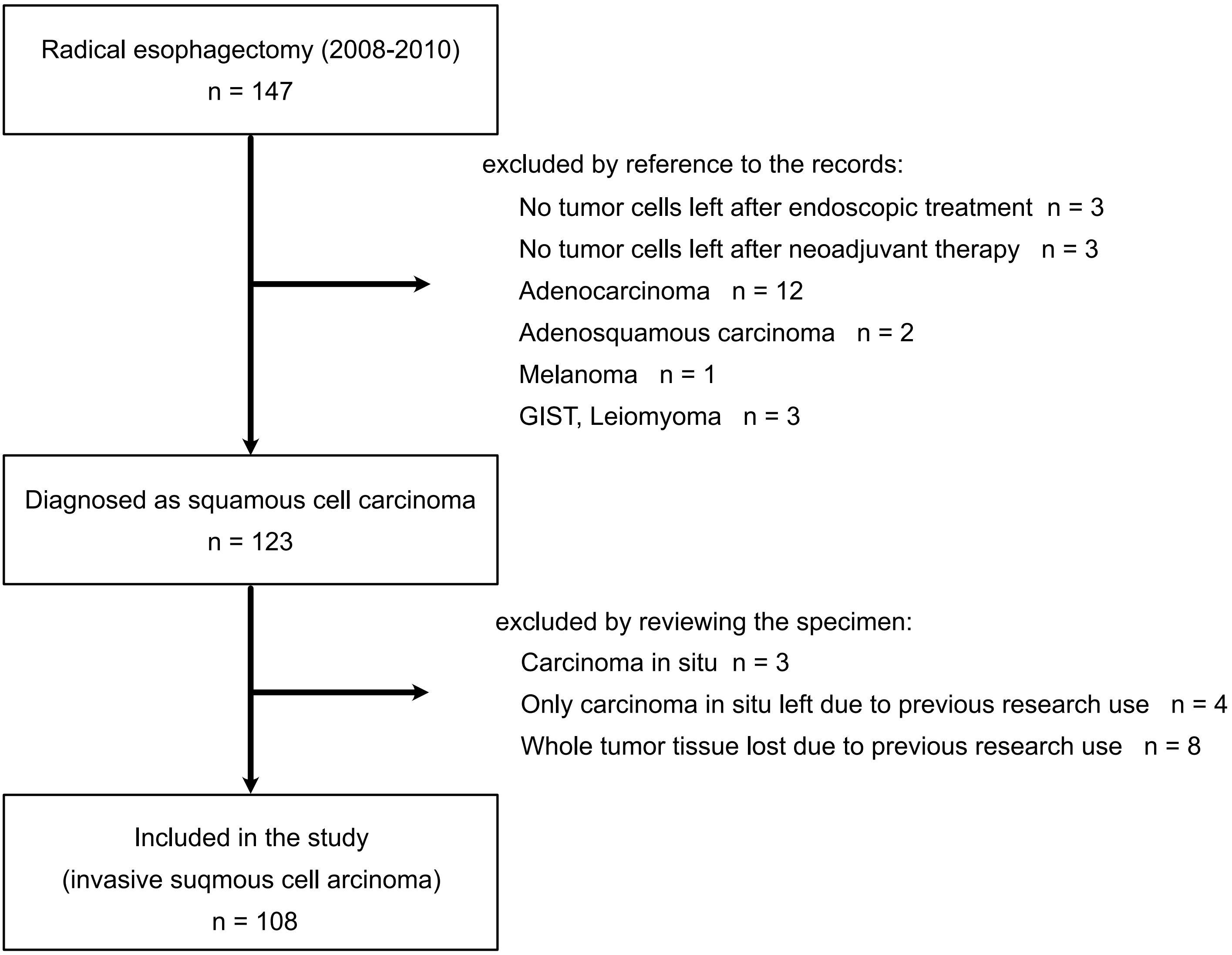

Fig. S1. Flow diagram of case selection.

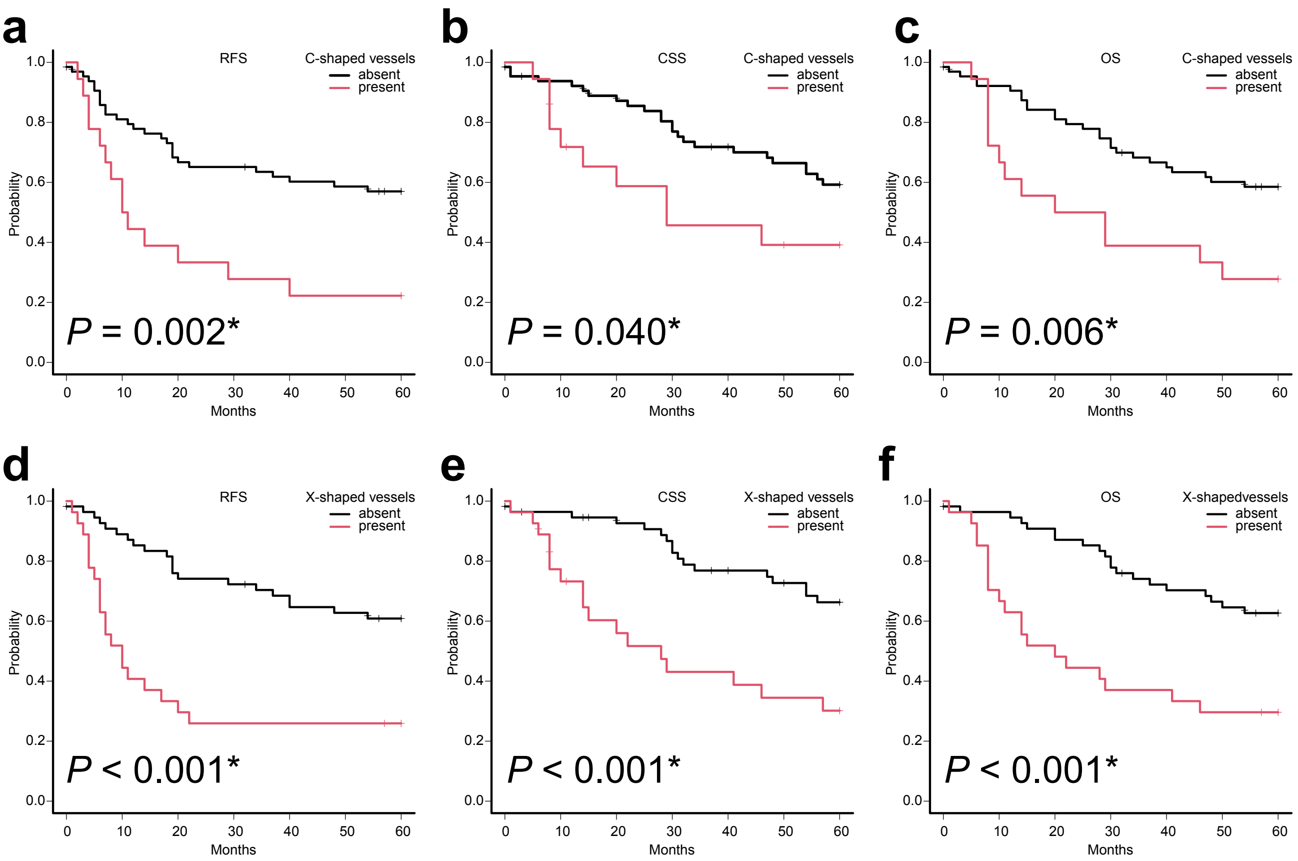

Fig. S2. Kaplan-Meier survival analysis on a population who did not receive neoadjuvant therapy in esophageal cancer (n = 83) . **(a-c)** 5-year RFS, CSS, and OS with C-shaped microvessels. **(d-f)** 5-year RFS, CSS, and OS with X-shaped microvessels.

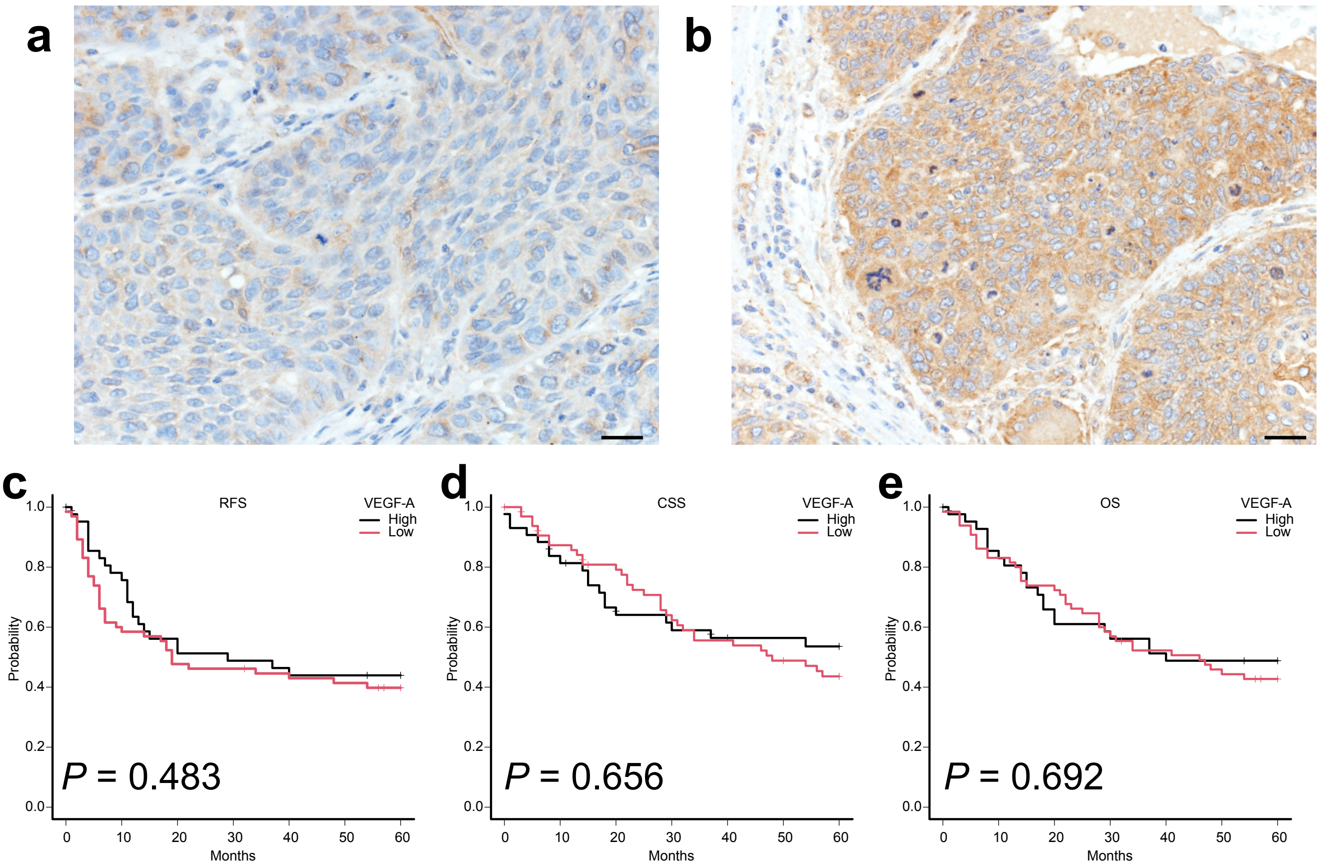

Fig. S3. VEGF-A expression throughout the tumor area and its association with prognosis. Cases were classified as either VEGF-A low or VEGF-A high based on a semi-quantitative score that considered both the proportion of positive cells and the staining intensity. **(a)** VEFG-A low. **(b)** VEGF-A high. **(c-e)** Kaplan-Meier analysis for 5-year relapse-free survival (RFS), cancer-specific survival (CSS), and overall survival (OS) with VEGF-A in 108 cases of esophageal cancer. Scale bars indicate 50μm.

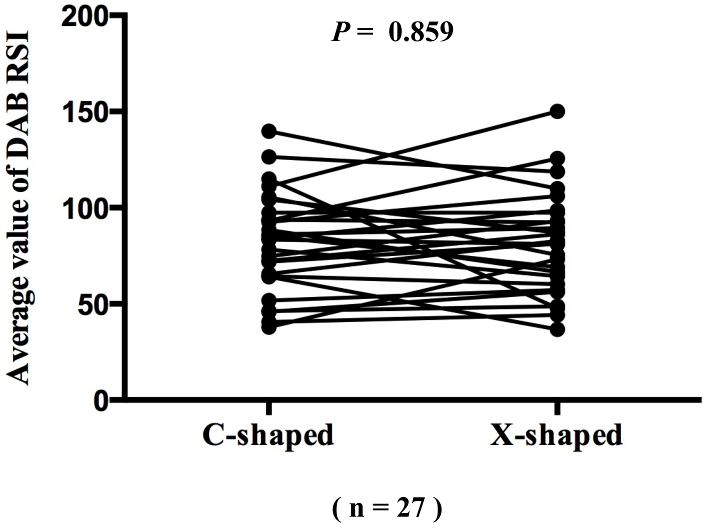

Fig. S4. Paired focal VEGF-A staining intensity in tumor cells surrounding C-shaped and X-shaped blood vessels. The average staining intensity in the surrounding tumor was compared between C-shaped and X-shaped microvesssels among cases where both types were present. The DAB staining intensities were measured as "mean gray value" and expressed as reciprocal staining intensities (RSI = 255 – mean gray value).
